# On the prior use of high-fidelity simulation to improve clinical trial development and implementation

**DOI:** 10.1017/cts.2025.10215

**Published:** 2026-01-22

**Authors:** Jeffrey Taekman

**Affiliations:** Professor Emeritus of Anesthesiology, Duke University School of Medicine, Durham, NC, USA

I read with great interest the recent article by Dadiz et al., *“Simulation as a Potential Tool for Successful Clinical Trial Initiation”* [1], which describes the use of simulation to identify latent barriers in trial start-up and optimize team-based implementation. The authors are to be commended for advancing this important conversation and documenting a process that will likely improve trial readiness across many domains.

In reviewing their work, I noted a close parallel to a body of research my team and I published between 2004 and 2010, in which we developed and evaluated the use of high-fidelity simulation not only for training research coordinators, but also as a systems-level tool to refine clinical trial protocols, clarify team roles, and proactively mitigate latent safety threats before real-patient enrollment.

Our early work – conducted in collaboration with the Duke Clinical Research Institute and Dr Robert Califf (former Commissioner of the U.S. Food and Drug Administration) – demonstrated:

- Improved study coordinator confidence and protocol readiness through simulation [2].
- The value of “walking through” a trial using simulation to identify operational pitfalls [3].
- Evidence of learning curves in protocol adherence, highlighting the importance of pre-implementation rehearsal [4].

While the current paper appears to have been developed independently, I write to provide this context for historical completeness and to encourage recognition of simulation as a field-tested methodology with a deeper legacy in clinical research operations than is often cited. I appreciate the authors’ open and collegial response to this point and their invitation to continue the conversation.

As interest in simulation grows across the translational science ecosystem, I hope this lineage is helpful to other investigators working at the intersection of protocol development, human performance, and team science.
